# Spatiotemporal assessment of spontaneous metastasis formation using multimodal *in vivo* imaging in HER2^+^ and triple negative metastatic breast cancer xenograft models in mice

**DOI:** 10.1371/journal.pone.0196892

**Published:** 2018-05-03

**Authors:** Inga B. Fricke, Raquel De Souza, Lais Costa Ayub, Giulio Francia, Robert Kerbel, David A. Jaffray, Jinzi Zheng

**Affiliations:** 1 TECHNA Institute for the Advancement of Technology for Health, University Health Network, Toronto, Ontario, Canada; 2 Institute of Biomaterials and Biomedical Engineering, University of Toronto, Toronto, Ontario, Canada; 3 Leslie Dan Faculty of Pharmacy, University of Toronto, Toronto, Ontario, Canada; 4 Biological Sciences Platform, Sunnybrook Research Institute, Department of Medical Biophysics, University of Toronto, Toronto, Ontario, Canada; 5 Radiation Medicine Program, Princess Margaret Cancer Centre, Toronto, Ontario, Canada; University of Tennessee Health Science Center, UNITED STATES

## Abstract

**Background:**

Preclinical breast cancer models recapitulating the clinical course of metastatic disease are crucial for drug development. Highly metastatic cell lines forming spontaneous metastasis following orthotopic implantation were previously developed and characterized regarding their biological and histological characteristics. This study aimed to non-invasively and longitudinally characterize the spatiotemporal pattern of metastasis formation and progression in the MDA-MB-231-derived triple negative LM2-4 and HER2^+^ LM2-4H2N cell lines, using bioluminescence imaging (BLI), contrast enhanced computed tomography (CT), fluorescence imaging, and 2-deoxy-2-[fluorine-18]fluoro-D-glucose positron emission tomography ([^18^F]FDG-PET).

**Material and methods:**

LM2-4, LM2-4H2N, and MDA-MB-231 tumors were established in the right inguinal mammary fat pad (MFP) of female SCID mice and resected 14–16 days later. Metastasis formation was monitored using BLI. Metabolic activity of primary and metastatic lesions in mice bearing LM2-4 or LM2-4H2N was assessed by [^18^F]FDG-PET. Metastatic burden at study endpoint was assessed by CT and fluorescence imaging following intravenous dual-modality liposome agent administration.

**Results:**

Comparable temporal metastasis patterns were observed using BLI for the highly metastatic cell lines LM2-4 and LM2-4H2N, while metastasis formed about 10 days later for MDA-MB-231. 21 days post primary tumor resection, metastases were detected in 86% of LM2-4, 69% of LM2-4H2N, and 60% of MDA-MB-231 inoculated mice, predominantly in the axillary region, contralateral MFP, and liver/lung. LM2-4 and LM2-4H2N tumors displayed high metabolism based on [^18^F]FDG-PET uptake. Lung metastases were detected as the [^18^F]FDG-PET uptake increased significantly between pre- and post-metastasis scan. Using a liposomal dual-modality agent, CT and fluorescence confirmed BLI detected lesions and identified additional metastatic nodules in the intraperitoneal cavity and lung.

**Conclusions:**

The combination of complementary anatomical and functional imaging techniques can provide high sensitivity characterization of metastatic disease spread, progression and overall disease burden. The described models and imaging toolset can be implemented as an effective means for quantitative treatment response evaluation in metastatic breast cancer.

## Introduction

Worldwide, breast cancer is one of the three most common cancers, and the most common in women. Over the course of their lifetime, one out of eight women will be diagnosed with breast cancer [1]. While early breast cancer without distant metastasis is a potentially curable disease, metastatic breast cancer is still considered incurable [2].

In the clinical setting, breast cancer is currently categorized into four subtypes: luminal A and B (estrogen receptor (ER) and/or progesterone receptor (PR) positive, human epidermal growth factor receptor 2 (ERBB2, HER2) negative), HER2 subtype (HER2^+^, ER^-^, PR^-^), and triple negative (TN; HER2^-^, ER^-^, PR^-^) breast cancer. Out of the different subtypes, TN breast cancer has an inferior prognosis, with an earlier development of distant metastases than other breast cancer subtypes [3].

Preclinical models of breast cancer frequently fail to recapitulate the clinical course of metastatic disease, resulting in drug development studies that are poorly predictive of clinical outcome [4]. This is mainly due to the use of (I) primary tumor models that do not lead to the formation of metastasis, and (II) metastatic models that do not recapitulate the actual process of metastasis formation.

Guerin et al. observed treatment efficacy of different antiangiogenic drugs in orthotopic primary TN breast cancer in mice, but not in the postsurgical metastatic state [5], confirming the findings from four failed randomized phase III clinical trials evaluating sunitinib in women with metastatic breast cancer [6]. A major difference in the biology of primary tumors and metastases that explains these findings is a phenomenon called vessel co-option. During their growth, primary tumors induce sprouting angiogenesis, while metastases can co-opt existing vasculature, making them less sensitive or insensitive to antiangiogenic therapy [7].

Mouse models that mimic breast cancer metastasis are often established by intravenous injection of tumor cells, not recapitulating the actual process of metastasis formation by seeding from a primary tumor. These models should be considered an organ colonization assay, rather than a metastatic model [8]. Therefore, models in which tumor cells are first implanted orthotopically and then spontaneously metastasize following surgical removal of the primary tumor, more closely recapitulate the process of metastasis formation, as well as the clinical presentation of advanced disease.

Metastases arise from cells of the primary tumor that gain the ability to undergo the metastatic cascade, comprised of local cell invasion, entry into the vasculature, extravasation and colonization at a distant site [9]. In order to generate cell lines with an increased metastatic potential, Munoz et al. established a protocol using two rounds of *in vivo* selection by orthotopic inoculation of tumor cells into immunocompromised mice, primary tumor removal, and isolation of cells from lung metastases [10]. Based on this protocol, various metastatic cell lines were created, one of which was the MDA-MB-231 derived TN LM2-4 cell line [10]. Through transduction of the LM2-4 cell line with HER2, the highly metastatic HER2^+^ LM2-4H2N cell line was produced, representing the HER2 subtype of breast cancer.

It has to be noted that the use of immunocompromised mice allows to generate models that use tumor cells of human origin, but do not account for the contribution of the (human) tumor microenvironment. The tumor microenvironment, composed of the immune system, tumor vasculature and lymphatics, fibroblasts, pericytes and sometimes adipocytes [11] can promote or repress tumor development.

Non-invasive, *in vivo* imaging techniques such as bioluminescence imaging (BLI), near-infrared (NIR) fluorescence, positron emission tomography (PET), and computed tomography (CT) enable us to follow the formation of metastasis longitudinally through relevant anatomical and functional descriptors, thereby overcoming the limitations of studies evaluating metastasis only by external inspection or necropsy at study endpoint. Specifically, these complementary imaging strategies either rely on the expression of reporter proteins (e.g. firefly luciferase (fLuc) for BLI), markers for metabolic activity (e.g. 2-deoxy-2-[fluorine-18]fluoro-D-glucose ([^18^F]FDG-PET) to monitor glucose metabolism), or tumor imaging contrast agents for fluorescence and CT, spanning a broad range of detection sensitivity and spatial resolution. [^18^F]FDG-PET, which is widely used for tumor imaging in the clinic, utilizes the enhanced glycolysis of cancer cells, leading to an increased uptake of [^18^F]FDG followed by its trapping in the cell due to hexokinase phosphorylation [12]. Based on its relatively low sensitivity in small lesions and low avidity in grade 1 cancers, [^18^F]FDG is mainly used in diagnosis and staging of advanced and metastatic disease [13,14].

In this study, we aimed to characterize the spatiotemporal pattern of metastasis formation in the two highly metastatic breast cancer cell lines LM2-4 and LM2-4H2N using longitudinal *in vivo* BLI, liposome contrast enhanced CT and fluorescence imaging, and [^18^F]FDG-PET.

## Material and methods

### Animal experiments

All animal experiments were approved by the University Health Network (UHN) Animal Care Committee and adhere to the ethical guidelines of the Canadian Council on Animal Care. Experiments were performed and reported in compliance with the ARRIVE guidelines. Female SCID mice (Ontario Cancer Institute, Toronto, ON, Canada) were housed at constant temperature (20°C) and 40% relative humidity under a 12 h light/12 h dark schedule and were given *ad-libitum* access to food and water. The study endpoint was reached when ≥ 20% of the tumor surface were ulcerated, mice lost ≥ 20% of their bodyweight or showed pronounced ascites. At study endpoint, mice were sacrificed by cervical dislocation under Isoflurane anesthesia. The detailed experimental schedule can be found in **S1 Fig**.

### Cell culture & tumor implantation

The LM2-4 (metastatic variant of MDA-MB-231; obtained at passage 6 in 2012) and the LM2-4H2N (metastatic variant of MDA-MB-231 with overexpression of HER2, established by HER2 transduction as described in Du Manoir et al. [15]; obtained at passage 16 in 2012) were kindly provided by Dr. Robert Kerbel (Sunnybrook Research Institute, Toronto, ON, Canada) and were fLuc^+^. The parental fLuc^+^ MDA-MB-231/Luc cells were purchased from Cedarlane Labs in 2012 (Burlington, ON, Canada). All three cell lines were cultured in RPMI1640 medium (Gibco, Carlsbad, CA, USA) supplemented with 10% fetal bovine serum (Gibco). Upon receipt, cell lines were confirmed negative for Mycoplasma contamination by the Department of Paediatric Laboratory Medicine (The Hospital for Sick Children, Toronto, ON, Canada) using a PCR-based test method. The cells were not further genetically authenticated. Cells were implanted 2–4 weeks after thawing. Female SCID mice (10 weeks of age) were inoculated with 4x10^6^ cells in 50 μl medium without supplements into their lower right inguinal mammary fat pad (MFP). Primary tumors were surgically resected two weeks post inoculation as previously described [10]. The surgical procedure was performed under Isoflurane anesthesia. Meloxicam (5 mg/kg) analgesia was administered before and at 24 and 48 hours post surgery.

### Primary tumor volume measurements

The volume of the primary tumor was determined prior to surgical removal using digital caliper. The following equation was used to calculate the tumor volume: calculated volume [mm^3^] = (π/6) x (length [mm] x (width [mm])^2^).

### Bioluminescence imaging (BLI)

Primary and metastatic tumor development was monitored longitudinally by *in vivo* bioluminescence imaging (BLI) using an IVIS Spectrum Imaging System (PerkinElmer, Waltham, MA, USA) and Living image 4.3.1 software. Mice were injected i.p. with 150 mg/kg D-Luciferin (Caliper Life Sciences, Hopkinton, MA, USA) 10 min before BLI acquisition (exposure time: 1–60 sec; field of view: D; subject height: 1.5 cm; binning: 8; f-stop: 1). 10 min was previously observed to be the peak time of the BLI signal in orthotopic MFP tumors. Regions of interest were drawn manually and BLI signal was quantified as total photon flux (photons/second).

### Computed tomography (CT)

Primary tumor volumes were quantified using a preclinical micro-computed tomography (μCT: Locus Ultra, GE Healthcare, Milwaukee, WI, USA). At 48 hours prior to the study endpoint, 200 μl of the CT/optical liposomal dual-modality contrast agent, CF800 [16], were administered i.v. and *in vivo* μCT images (80 kVp, 50 mA) were acquired before and at 3, 24, and 48 hours post liposome contrast agent injection. CT images were analyzed using the Inveon^TM^ Research Workplace software (Siemens Healthcare, Erlangen, Germany). As a non-targeted liposomal contrast agent, CF800 accumulates in the tumor based on the enhanced permeability and retention (EPR) effect [16]. Based on the variability in perfusion, permeability, and degree of EPR effect in different tumor sites and different tumor models, the contrast that can be achieved with CF800 is variable. The images shown in this publication were displayed to represent the achieved contrast as good as possible.

### Fluorescence imaging

Forty-eight hours after the administration of CF800, *ex vivo* 2-D NIR fluorescence imaging (excitation = 785 nm, emission = 820 nm) of the animals with their abdominal skin opened was performed using the Maestro™ system (Cambridge Research & Instrumentation, Woburn, MA, USA) in order to visualize metastatic lesions at the study endpoint. As non-invasive fluorescence imaging is less sensitive than BLI, imaging without opening the abdominal skin did not give sufficient information to characterize the metastatic spread.

### [^18^F]FDG positron emission tomography (PET)

2-deoxy-2-[fluorine-18]fluoro-D-glucose ([^18^F]FDG)-PET was acquired in order to assess the metabolic activity of the primary tumors 3 days before resection (on day 11 post tumor cell inoculation) and of the metastatic lesions at 32 days after the initial tumor cell inoculation (18 days post primary tumor resection). [^18^F]FDG was produced by the Centre for Probe Development and Commercialization (CPDC; Hamilton, ON, Canada). Mice were fasted overnight and 10-minute emission PET scans were performed at 1.04 ± 0.04 h post i.v. injection of 9.42 ± 2.31 MBq [^18^F]FDG (0.55 ± 0.12 MBq/g) using a triple mouse imaging bed (Minerve, Esternay, France) on a Focus 220 preclinical PET scanner (Siemens), followed by an 8-minute ^57^Co transmission scan for attenuation and scatter correction. After the PET scan, CT imaging was acquired with the animals in the same position by transfer the imaging bed to the μCT scanner. Before [^18^F]FDG injection, a drop of blood was taken close to the tip of the tail and blood glucose levels were measured using a blood glucose meter (Contour^®^, Bayer HealthCare LLC, Mishawaka, IN, USA). Average blood glucose levels were 5.8 ± 1.8 mmol/l and 6.0 ± 1.1 mmol/l for the two imaging days across all 12 and 11 animals, respectively. PET reconstruction was performed using a 3D ordered subset expectation maximization (OSEM3D) and maximum a posteriori (MAP) algorithm with two iterations of OSEM3D and 18 iterations of MAP. The reconstructed image dataset has a voxel size of 0.316 mm x 0.316 mm x 0.796 mm. Images were analyzed using the Inveon^TM^ Research Workplace software. Volumes of interest (VOIs) were drawn manually for primary tumors and metastatic lesions. To quantify muscle uptake, a standard VOI (19.9 ± 0.71 mm^3^) was placed on the contralateral femoral muscle.

### Statistical analysis

Statistical analysis was performed using GraphPad Prism 7 (GraphPad Software, La Jolla, CA, USA). A *P*-value of < 0.05 was considered significant. Where applicable, paired/unpaired *t*-tests or one-way ANOVA were performed. Two-way ANOVA with multiple comparisons (Sidak) was used to analyze lung [^18^F]FDG uptake before and after metastasis formation. Correlation analysis was performed using Pearson correlation. Linear regression lines were fitted through the origin.

## Results

### Primary tumor growth

**Fig 1:**
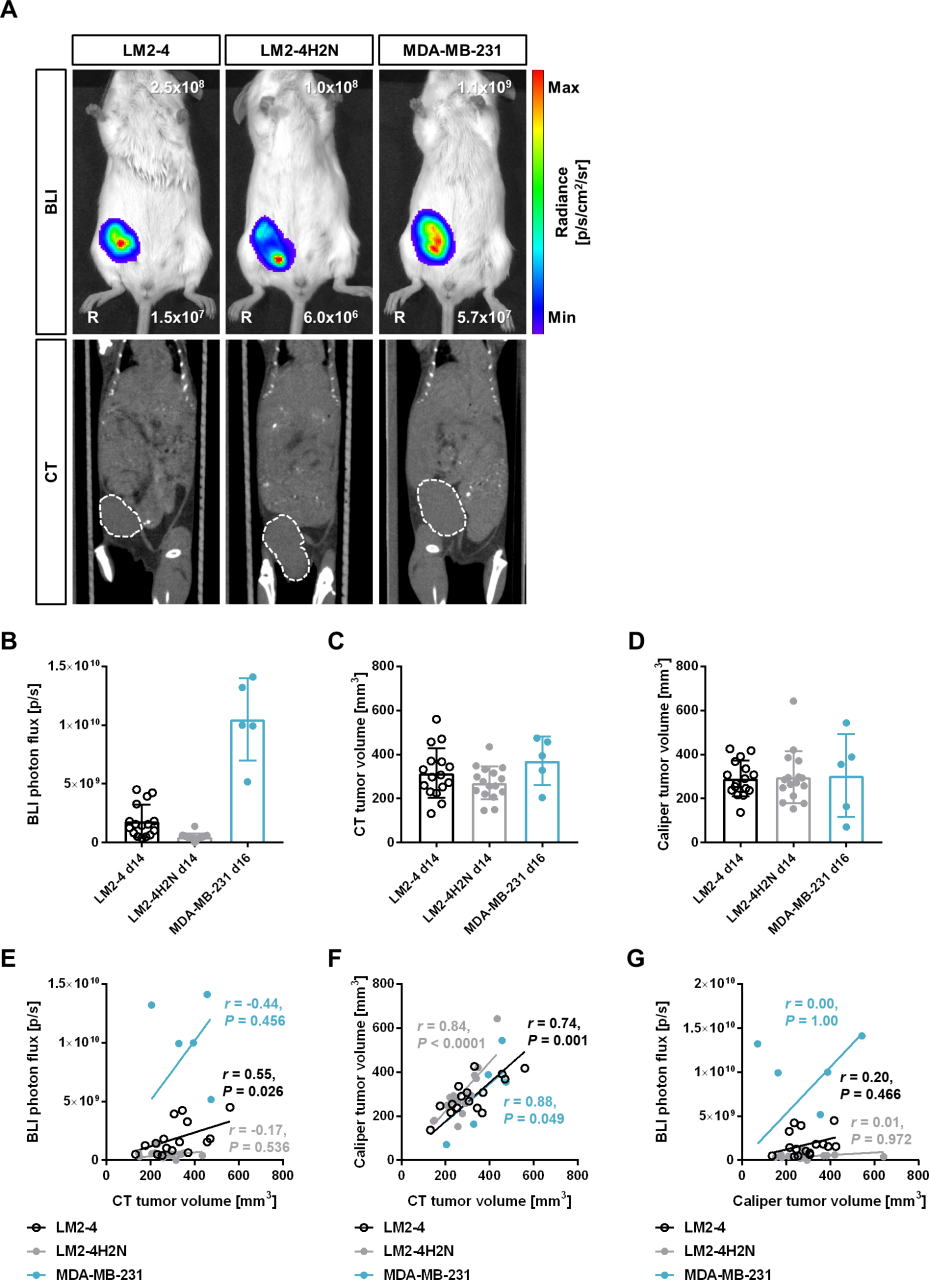
Characterization of primary tumor growth for orthotopically implanted LM2-4, LM2-4H2N and MDA-MB-231 cells. (A) Representative BLI and CT images of primary tumors before resection. Dashed lines outline the tumor contour. R: right. (B) Quantification of primary tumor BLI signal. (C-D) Quantification of primary tumor volume before resection based on CT (C) and caliper measurements (D). (E-G) Correlation between CT tumor volume and BLI signal (E), CT tumor volume and caliper measurements (F), and caliper measurements and BLI signal (G).

Following inoculation of LM2-4, LM2-4H2N, and MDA-MB-231 tumor cells into the lower right MFP, primary tumor growth was monitored by BLI, CT, and caliper measurements. Representative BLI and CT images of the primary tumors before resection are shown in **Fig 1A**. Primary tumors grew within the intended MFP and reached a size of 304.0 ± 100.6 mm^3^ at the time of resection (day 14 post inoculation for LM2-4 and LM2-4H2N, and day 16 post inoculation for MDA-MB-231). Based on a different expression of fLuc in the transfected cell lines, the BLI signal varied within approximately one order of magnitude between LM2-4, LM2-4H2N, and MDA-MB-231 (**Fig 1B**) despite the three groups having similar tumor volumes as determined by CT (LM2-4: 315.8 ± 112.2 mm^3^, *n* = 16; LM2-4H2N: 271.1 ± 75.21 mm^3^, *n* = 16; MDA-MB-231: 371.7 ± 109.8 mm^3^, *n* = 5; *P* = 0.12, One-way ANOVA, **Fig 1C**) and caliper measurement (LM2-4: 291 ± 80.63 mm^3^, *n* = 16; LM2-4H2N: 297.3 ± 118.2 mm^3^, *n* = 16; MDA-MB-231: 304.3 ± 187.9 mm^3^, *n* = 5; *P* = 0.97, One-way ANOVA, **Fig 1D**). The highest BLI photon flux was observed for MDA-MB-231 (1.1x10^10^ ± 3.5x10^9^ p/s), followed by LM2-4 (1.8x10^9^ p/s ± 1.4x10^9^), while LM2-4H2N (5.0x10^8^ ± 2.9x10^8^ p/s) displayed the lowest BLI signal (**Fig 1B**). The BLI signal showed a higher variability than the tumor volume measurements by CT (**Fig 1C**) or caliper (**Fig 1D**). Tumor volumes at resection, as determined by CT or caliper, were not statistically different between the three models. A significant correlation between BLI photon flux and CT tumor volume was only observed for the LM2-4 model (**Fig 1E**; *r* = 0.55, *P* = 0.026, Pearson), while CT tumor volume and caliper measured tumor volume measurements correlated positively for all models (**Fig 1F**; LM2-4: *r* = 0.74, *P* = 0.001; LM2-4H2N: *r* = 0.84, *P* < 0.0001; MDA-MB-231: *r* = 0.88, *P* = 0.049; Pearson). No correlation was found between BLI photon flux and caliper measured tumor volume (**Fig 1G**; LM2-4: *r* = 0.20, *P* = 0.466; LM2-4H2N: *r* = 0.01, *P* = 0.972; MDA-MB-231: *r* = 0.00, *P* = 1.00; Pearson).

### Timing of metastasis formation

**Fig 2:**
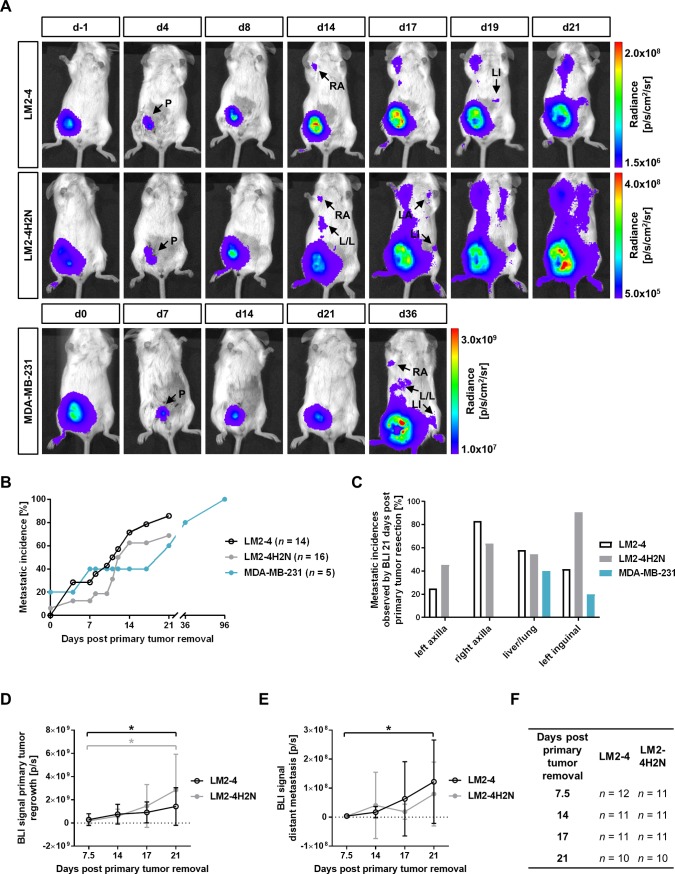
Spatiotemporal pattern of metastasis formation after removal of the primary LM2-4, LM2-4H2N and MDA-MB-231 tumor. (A) Representative images of BLI assessment of metastatic burden following removal of the primary LM2-4, LM2-4H2N, and MDA-MB-231 tumor. Arrows illustrate primary regrowth (P) and metastatic regions: left inguinal region (LI), left and right axillary region (LA/RA), liver/lung region (L/L). (B) Metastatic incidences excluding primary regrowth detected by BLI over time after removal of the primary tumor. (C) Metastatic incidences detected by BLI on day 21 post primary tumor resection for different anatomical locations. (D-E) BLI signal from the primary tumor regrowth (D) and from distant metastasis (E) for all animals that eventually develop metastasis. *P < 0.05, *t*-test. (F) Numbers of animals included in panel D & E.

In order to non-invasively follow metastasis formation following primary tumor resection, longitudinal *in vivo* BLI was performed (**Fig 2A**). The first metastatic events, not including regrowth at the site of the primary tumor removal, were observed between day 4 and day 21 post primary tumor resection for LM2-4, between day 0 and day 21 for LM2-4H2N, and between day 0 and 96 for MDA-MB-231. Two out of 14 mice of the LM2-4 group, 5 out of 16 mice of the LM2-4H2N group, and 0 out of 5 mice of the MDA-MB-231 group did not develop distal metastatic disease (other than primary tumor regrowth) during the duration of the experiment (21–24 days post primary removal for LM2-4/LM2-4H2N and 96 days post primary removal for MDA-MB-231; **Fig 2B**). We observed about 23% more metastases in the LM2-4 and LM2-4H2N model on day 21 than in the MDA-MB-231 model on day 96, despite the longer follow-up time for MDA-MB-231. In both the LM2-4 and LM2-4H2N groups, one mouse was excluded from the study due to formation of ascites, and two mice in the LM2-4 group died around the time of primary tumor resection due to advanced disease. A comparable temporal pattern of metastasis formation was observed using BLI for the two highly metastatic cell lines (median time to develop a first metastasis of 10.5–12 days post primary tumor resection), while metastasis formed about 10 days later for the parental MDA-MB-231 cell line (median time to develop a first metastasis of 21 days, **Fig 2B**). The first regions to develop metastasis in the LM2-4 model were the right axillary region and the liver/lung region (starting at day 4 following primary tumor resection), followed by the left inguinal region (starting at day 8, **Fig 2A**).

In the LM2-4H2N model, one out of 16 animals already showed BLI signal in the left and right axillar region at the time of primary tumor removal, while metastasis in other locations appeared at similar times compared to LM2-4 (liver/lung region day 4, left inguinal region day 4; all post primary tumor removal). At 21 days post primary tumor removal, LM2-4 and LM2-4H2N inoculated animals showed high incidence rates (54.5–83.3%) for metastasis in the right axillary and liver/lung region, while incidence rates for the left axillary region (25.0% for LM2-4 and 45.5% for LM2-4H2N) and left inguinal region (41.7% for LM2-4 and 90.9% for LM2-4H2N) were more variable (**Fig 2A and 2C**). In the MDA-MB-231 model, metastasis could only be observed in the liver/lung region at 21 days post primary tumor removal (**Fig 2C**). All models showed high rates of regrowth (80–100%) at the primary tumor location, first detectable between day 4 and 7 post primary tumor removal (**Fig 2A**).

The tumor regrowth at the primary location and the formation of distant metastasis were quantified for LM2-4 and LM2-4H2N based on BLI photon flux from the right MFP region (primary regrowth, **Fig 2D**) and the rest of the mouse (signal from distant metastasis = whole body signal–primary tumor regrowth signal, **Fig 2E**). The number of animals analyzed for each model is summarized in **Fig 2F**. The MDA-MB-231 tumors were not included in this analysis as too few animals displayed metastatic BLI signal on day 21 post primary tumor removal. The signal from the primary tumor location increased significantly between day 7.5 and day 21 post primary tumor removal in the LM2-4 (day 7.5: 2.9x10^8^ ± 5.1x10^8^ p/s, *n* = 12; day 21: 1.4x10^9^ ± 1.6x10^9^ p/s, *n* = 10; *P* = 0.02, *t*-test) and LM2-4H2N (day 7.5: 1.8x10^8^ ± 2.7x10^8^ p/s, *n* = 11; day 21: 2.9x10^9^ ± 3.1x10^9^ p/s, *n* = 10; *P* = 0.01, *t*-test) model (**Fig 2D**). In the same time interval, an increase in BLI signal from the rest of the mouse as measure for development of metastasis **(Fig 2E)** was observed for LM2-4 (day 7.5: 3.8x10^6^ ± 6.8x10^6^ p/s, *n* = 12; day 21: 1.2x10^8^ ± 1.4x10^8^ p/s, *n* = 10; *P* = 0.03, *t*-test), but did not reach statistical significance for LM2-4H2N (day 7.5: 3.1 x10^6^ ± 4.3 x10^6^ p/s, *n* = 11; day 21: 8.0x10^7^ ± 1.1x10^8^ p/s, *n* = 10; *P* = 0.05, *t*-test). As the metastatic burden might be related to the primary tumor size upon resection, we also calculated the metastatic burden/primary tumor BLI signal ratio, which again showed a significant increase over time for LM2-4, but did not reach significance for LM2-4H2N (**S2 Fig**). As the primary tumors displayed a very similar volume upon resection (LM2-4: 315.8 ± 112.2 mm^3^; LM2-4H2N: 271.1 ± 75.2 mm^3^), we did not observe an impact of the normalization of the BLI signal from distant metastasis using the primary tumor volume upon resection. Moreover, we could not detect a correlation between primary tumor volume before resection and BLI signal from distant metastasis at study endpoint for both cell lines (LM2-4: *r* = 0.44, *P* = 0.199, *n* = 10; LM2-4H2N: *r* = 0.05, *P* = 0.888, *n* = 10; Pearson).

### Metabolic activity of primary and metastatic lesions assessed by [^18^F]FDG-PET/CT

**Fig 3:**
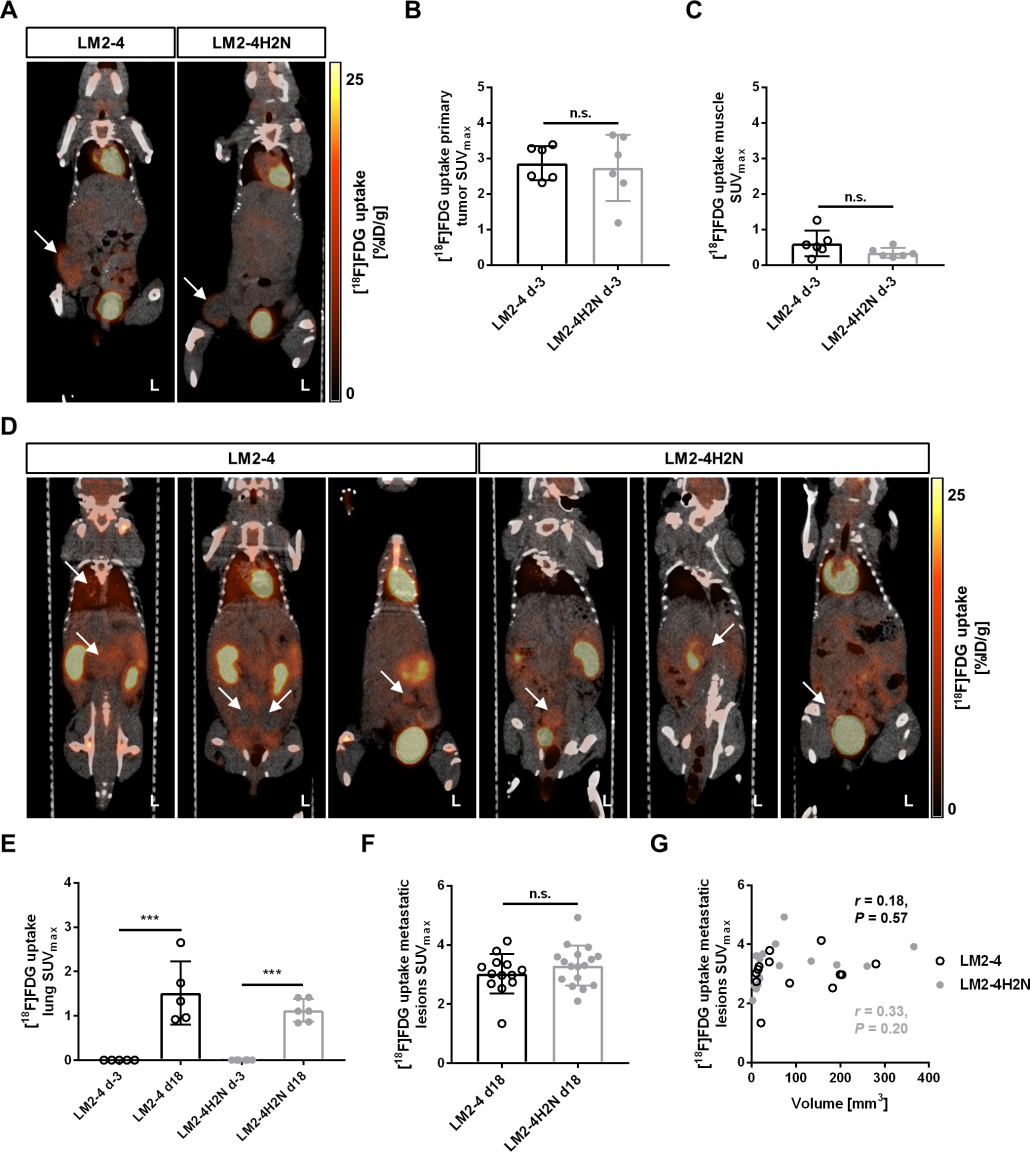
[^18^F]FDG-PET of metabolic activity of primary tumors and metastasis of LM2-4 and LM2-4H2N cells. (A) Representative images of [^18^F]FDG uptake in primary tumors of LM2-4 and LM2-4H2N at 11 days post inoculation. Arrows indicate the tumor location. (B) [^18^F]FDG uptake in SUV_max_ for the primary tumor. (C) [^18^F]FDG uptake in SUV_max_ for the muscle. (D) Representative images of [^18^F]FDG uptake at different metastatic sites (labelled with arrows) on day 18 post primary tumor removal. (E-F) [^18^F]FDG uptake in SUV_max_ in lung (E) and in metastatic lesions (F) at 18 days post primary tumor removal. (G) Correlation between [^18^F]FDG uptake in SUV_max_ and metastatic nodule volume. L: left. **P < 0.01, ***P < 0.001, Two-way ANOVA/*t*-test.

In order to assess possible differences in metabolic activity of primary tumors and metastatic lesions in the LM2-4 and LM2-4H2N models, [^18^F]FDG-PET was performed on days 11 and 32 post tumor inoculation (corresponding to days -3 and 18 post primary tumor removal; **Fig 3**). LM2-4 and LM2-4H2N inoculated mice showed a comparable [^18^F]FDG uptake in their primary tumor lesions (SUV_max, LM2-4_ = 2.87 ± 0.20, *n*_LM2-4_ = 6, SUV_max, LM2-4H2N_ = 2.74 ± 0.38, *n*_LM2-4H2N_ = 6, *P* = 0.77, *t*-test, **Fig 3A and 3B**), as well as in the muscle (SUV_max, LM2-4_ = 0.61 ± 0.15, *n*_LM2-4_ = 6, SUV_max, LM2-4H2N_ = 0.35 ± 0.06, *n*_LM2-4H2N_ = 6, *P* = 0.13, *t*-test, **Fig 3A and 3C**). A significant increase (280–300 fold) in lung uptake between day -3 and 18 post primary tumor removal was observed in both tumor models (SUV_max, LM2-4, d-3_ = 0.005 ± 0.001, SUV_max, LM2-4, d18_ = 1.52 ± 0.71, *n* = 5, *P* = 0.0002; SUV_max, LM2-4H2N, d-3_ = 0.004 ± 0.001, SUV_max, LM2-4H2N, d18_ = 1.13 ± 0.25, *n* = 5, *P* = 0.0009, Two-way repeated measures ANOVA with multiple comparisons (Sidak), **Fig 3A, 3D and 3E**), while no significant difference in lung uptake was observed between LM2-4 and LM2-4H2N on days -3 and 18 (*P* = 0.2322, Two-way repeated measures ANOVA). The low [^18^F]FDG uptake values measured on the first day indicate that there was no lung metastasis prior to primary tumor removal. On day 18 post primary tumor resection, a mean SUV_max_ [^18^F]FDG uptake of 3.02 ± 0.67 and 3.30 ± 0.68 was observed in the metastatic lesions and the primary tumor regrowth of LM2-4 and LM2-4H2N (**Fig 3F**), respectively. [^18^F]FDG-detectable metastatic lesions were observed in the lower abdomen in proximity to the primary tumor regrowth and in the middle abdomen close to the liver/lung region, but not in the axillary regions. In both models, the SUV_max_ [^18^F]FDG uptake did not correlate with the size of the lesions (LM2-4: *r* = 0.18, *P* = 0.57; LM2-4H2N: *r* = 0.33, *P* = 0.20, Pearson; **Fig 3G**), nor was the uptake related to the anatomical location of the lesion.

### Imaging of breast cancer metastasis using contrast-enhanced CT & *ex vivo* fluorescence imaging and histologic confirmation of nodules detected by *in vivo* imaging

**Fig 4:**
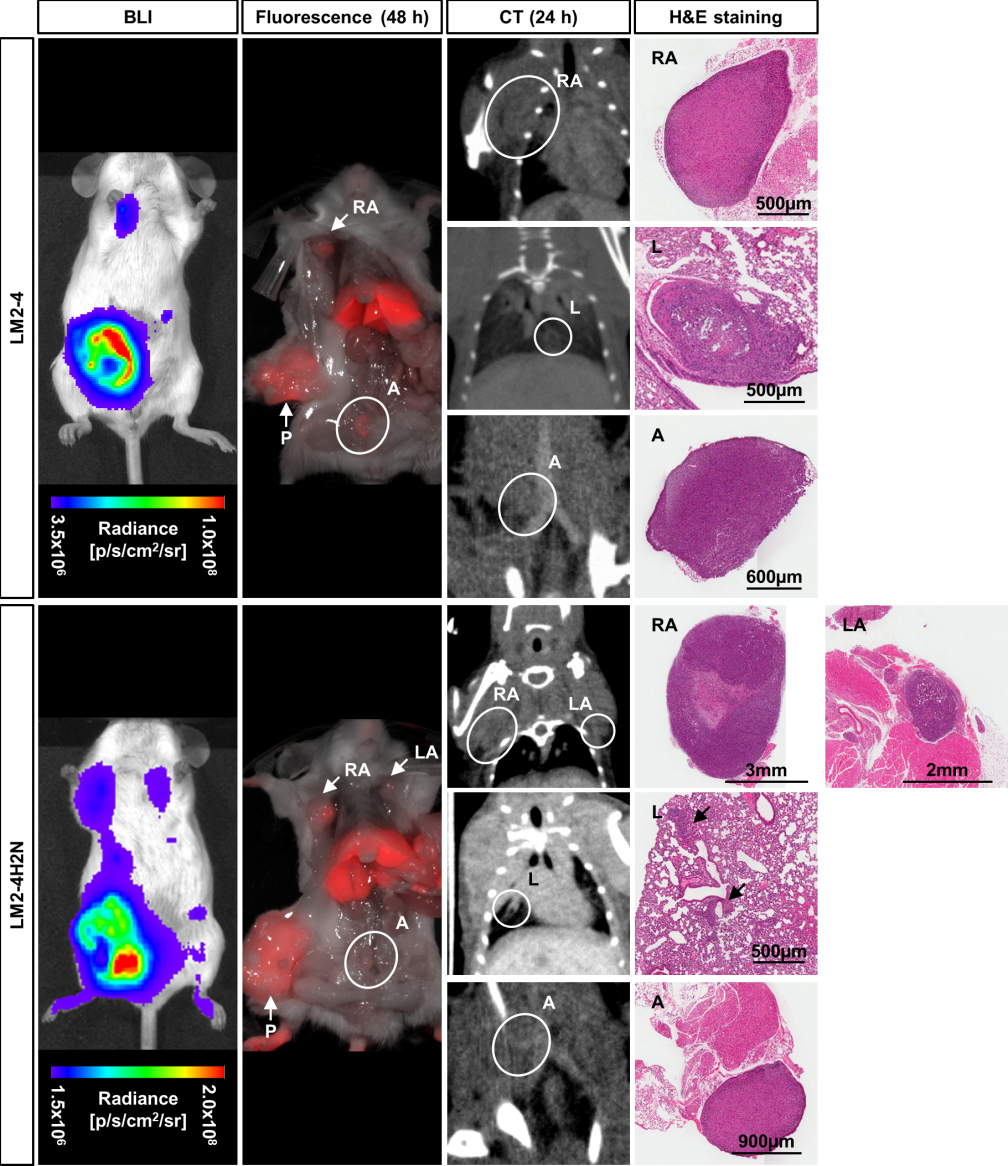
*In vivo* and *ex vivo* imaging of breast cancer metastasis at study endpoint for LM2-4 and LM2-4H2N. Imaging of breast cancer metastasis using bioluminescence imaging (BLI), and *ex vivo* fluorescence and *in vivo* CT imaging at 48h or 24h, respectively, following injection of the dual-modality contrast agent. Metastatic nodules were confirmed by H&E staining. RA/LA: right/left axillary nodule, P: primary tumor regrowth, L: lung, A: abdominal nodule.

With its relatively high sensitivity, BLI can assess the temporal pattern of metastasis formation, while its lack in spatial resolution hinders the identification of small solitary nodules. Because of its high spatial resolution, we used CT following administration of a liposomal dual modality CT/optical contrast agent passively targeted to tumor sites [16]. The liposomal encapsulation results in a long circulating imaging agent, expanding the tumor visualization window to 48 hours [16] (**S3 Fig**). We previously determined that at least 24 hours is needed for the liposome agent to reach a good CT signal-to-background ratio at tumor sites [16]. The main signal enhancement is observed in the tumor margins. We used the 24h CT data set to confirm metastatic nodules in the regions which displayed BLI signal, including the primary tumor regrowth (**S3 Fig**) and axillary nodules (**Fig 4**). In addition, small solitary nodules not detected by BLI could be identified in various locations, including the lung and lower peritoneum (**Fig 4**). *Ex vivo* fluorescence imaging at 48h post contrast agent injection and histology confirmed the presence of non BLI-detectable nodules.

## Discussion

In this study, we aimed to characterize primary tumors and the spatiotemporal pattern of spontaneous metastasis formation in two highly metastatic breast cancer xenograft models using BLI, CT, fluorescence imaging, and [^18^F]FDG-PET, thereby providing a non-invasive toolset to monitor and quantify metastatic disease spread, progression and response to therapy, highly valuable for future drug development studies.

We observed a similar growth of primary LM2-4, LM2-4H2N, and MDA-MB-231 tumors, while following primary tumor resection, both LM2-4 and LM2-4H2N models developed metastasis earlier (roughly in half the time) and in higher numbers (23% more nodules) than the parental MDA-MB-231 model. The main sites of metastasis were the axillary regions, liver/lung, and left MFP for LM2-4 and LM2-4H2N, and liver/lung and left MFP for MDA-MB-231. As determined by [^18^F]FDG-PET, LM2-4 and LM2-4H2N primary tumors and metastatic lesions were highly metabolically active. The post-metastasis scan showed a significant increase (280–300 fold) in [^18^F]FDG uptake in the lung compared to pre-metastasis for LM2-4 and LM2-4H2N. Liposome contrast enhanced CT and *ex vivo* fluorescence at study endpoint confirmed all BLI detectable tumor lesions and identified additional metastatic nodules, including those in the intraperitoneal cavity and the lung.

Previous studies on metastasis formation from MDA-MB-231 [17,18], LM2-4 [10,17], and LM2-4H2N [19] xenografts in SCID mice mainly relied on the evaluation of metastasis formation at study endpoint, lacking information about its spatiotemporal pattern. Looking at the primary MFP tumors, we observed a comparable growth for all three cell lines, which was previously described for MDA-MB-231 and LM2-4 [17]. As the extend of metastasis formation might be related to the primary tumor size upon resection, it is crucial to resect primary tumors at a standardized volume. For the parental MDA-MB-231 cell line, we observed a delayed formation of metastasis in comparison to the LM2-4 and LM2-4H2N cell line, with metastasis mainly forming in the liver/lung region. In line with our findings, Milsom *et al*. described the lung as the most common location for metastasis (75%) in the MDA-MB-231 cell line, followed by lymph nodes (37.5%) [17]. The LM2-4H2N cell line was generated from the LM2-4 cell line by transfection with HER2 [10] with the intent to generate a cell line to test HER2-targeted treatments, and displayed a similar behaviour *in vivo*. In both these cell line derived tumor models, we observed pronounced metastatic disease at 3 weeks post primary tumor resection with metastasis in liver/lung, the axillary regions, and the contralateral MFP, with incidence rates comparable to those previously described for the orthotopic LM2-4 model [17]. Clinical studies describing the pattern of metastasis formation in TN breast cancer found higher rates of recurrence in visceral organs and soft tissue, but lower rates in bone, compared to hormone receptor positive disease [20]. Lin *et al*. described that the most common sites of involvement upon diagnosis of TN breast cancer metastatic disease were lung, liver, bone, and breast or chest wall (41%, 29%, 24% and 22% of patients, respectively) [21]. Except for the lack of bone metastasis, this pattern is comparable to the pattern of metastasis formation in the highly metastatic LM2-4 and LM2-4H2N models. The absence of bone metastasis is a common feature of xenograft models using human breast cancer cells in mice, as breast cancer cells exploit a strong species-specific osteotropism [8,22]. Several publications reported a preference of TNBC to metastasize to lung, while HER2^+^ breast cancer preferentially spreads to the liver [23–25]. Due to the low spatial resolution and missing anatomical information of BLI images, we could not clearly distinguish between lung and liver signal in the BLI analysis. However, no significant difference in [^18^F]FDG signal in the lung was observed between LM2-4 and LM2-4H2N, indicating that the artificially constructed LM2-4H2N cell line does not represent the metastatic spread observed HER2^+^ breast cancer in humans. Further along the disease development, 46% of TN breast cancer patients were described to develop central nervous system (CNS) metastasis [21]. This high incidence rate for brain metastasis is not recapitulated in the metastatic models described in this study and was not observed in previous studies [17]. It was previously proposed that the high incidences in CNS metastasis observed in the clinical setting arise from a prolonged control of local and visceral metastatic disease by chemotherapy, giving microscopic metastasis sufficient time to develop, and the fact that tumor cells find a sanctuary site in the brain due to the limited permeability of the blood brain barrier to most chemotherapy agents [26]. This effect is well described in trastuzumab-treated HER2^+^ breast cancer patients [27]. A similar increased incidence of brain metastasis following chemotherapy was also described in the preclinical setting in long-term surviving animals in a mouse model of melanoma metastatic disease [28] and following trastuzumab treatment in the LM2-4H2N model [19]. In a separate study performed by our group, a brain metastatic lesion was indeed observed in the LM2-4 model on day 77 post primary tumor removal (56 days later than the study endpoint for this investigation), after the animal received weekly chemotherapy for 9 weeks [29].

It has to be noted that the strong primary tumor regrowth observed in this model might generate metastasis not originating from the original tumor, but the primary tumor regrowth. Moreover, different rates of regrowth might contribute to the difference in metastatic sites and speed of metastatic development between the more metastatic and the parental cell line.

In summary, both the metastatic LM2-4 and LM2-4H2N cell lines display a metastatic pattern that is in line with reports on the metastatic pattern in triple negative breast cancer models and patients. The HER2 transduction of the LM2-4 cell line generating the LM2-4H2N cell line does not alter its metastatic pattern and should only be considered as a tool to enable testing of HER2-targeted therapies, but not as a model for HER2^+^ metastatic breast cancer.

BLI is a relatively fast, easy, and cost-effective modality to longitudinally follow metastatic spread in mice. However, it is a semi-quantitative method, which requires highly standardized protocols and careful data acquisition and analysis. As light travels through tissues, it is absorbed and scattered depending on the type and thickness of overlying tissue, resulting in a photon flux that is not necessarily correlated with the number of fLuc expressing cells. Positioning of the animal in ventral or dorsal position will lead to different quantification results. In this study, we found that BLI photon flux from the primary tumor and the tumor volume measured by CT correlated significantly only for the LM2-4 cell line. This observation could be related to a more homogenous growth in terms of tumor shape and depth location. In addition, the higher fLuc expression in MDA-MB-231 might lead to bigger variances in BLI signal. Additional factors influencing the BLI signal are the availability of ATP and oxygen, making the BLI signal vulnerable to changes in vascularization and processes like apoptosis and necrosis. In fact, we observed necrosis in 20–30% of the primary tumors upon resection. The regrowth of the primary tumor introduces another challenge to the quantification of the metastatic spread, as a weak signal from small metastasis might not be detected due to the stronger signal from the primary tumor regrowth. To account for this, future studies should consider blocking the primary tumor regrowth signal in order to better detect metastasis.

As an alternative, the i.v. injection of breast cancer cells might be a valuable model to follow metastasis formation using BLI without confounding effects from a primary tumor regrowth. In a separate study from our group, 1x10^6^ LM2-4 cells were injected i.v. in female SCID mice and cells were detected in the lung immediately after injection. Cells then cleared from the lung and first BLI-detectable metastasis were observed from day 9 on in the brain, followed by metastasis in multiple locations, including lymph nodes, lung, and liver. Study endpoint was reached at approx. 4 weeks post tumor cell injection due to severe metastatic disease (oral communication Dr. M. Ventura, University Health Network, Toronto, ON, Canada). The i.v. injection leads to accumulation of cells in the lungs early after injection, followed by very extensive lung metastasis in the later disease course which cannot directly be compared to the diffuse lung metastasis we observed in the model using orthotopic implantation followed by primary tumor resection. This model allows to study metastasis formation by BLI without the interfering signal from the primary tumor regrowth, but introduces new challenges as the strong lung signal hampers the detection of metastasis in the axillary regions. In addition, it has to be noted that i.v. injection of tumor cells does not replicate the actual process of seeding from a primary tumor and the course of metastasis formation starting in the brain does not replicate the course of metastatic disease in TN breast cancer in humans.

Due to its relatively low spatial resolution and 2D nature, BLI is unable to resolve single nodules in a cluster of nodules or close to a source of high BLI signal (e.g. the primary tumor regrowth). For the same reason, the detection of micrometastasis evenly distributed over the lung is challenging with BLI.

Therefore, we evaluated [^18^F]FDG-PET and liposomal contrast enhanced CT imaging in their ability to complement the BLI imaging to provide additional functional and anatomical information on metastatic lesions. [^18^F]FDG-PET showed to be especially useful to detect the presence of many small, but widespread metastatic lesions in the lung. The high uptake in the heart hinders the quantification, only allowing representative regions of interest further away from the heart for quantification. Previous studies underlined the value of [^18^F]FDG in the detection of lung metastasis and described the identification of bigger lung lesions based on [^18^F]FDG which were not located in direct proximity to the heart [30,31]. Using [^18^F]FDG-PET, we observed metastatic lesions in the lower and middle abdomen that would not be distinguishable from signal from the primary tumor regrowth or liver/lung signal using BLI. Smaller lesions in the left and right axillary regions could not be detected by [^18^F]FDG PET, indicating that this method is not sensitive enough to be used alone to characterize metastatic spread. Using liposomal contrast enhanced CT imaging, we could leverage the high spatial resolution of CT to identify lung nodules even in close proximity to the heart, which could not be detected using [^18^F]FDG-PET or BLI. Additionally, better discrimination of nodule groups and single nodules could be achieved in the intraperitoneal cavity following the injection of the liposome imaging agent and imaging using CT *in vivo* and NIR fluorescence post-mortem. Even though CF800 allows for intraoperative fluorescence imaging facilitating tumor resection, *in vivo* NIR fluorescence imaging was not performed as due to background signal and signal attenuation from overlying tissues, *in vivo* fluorescence imaging was not as sensitive as BLI in detecting metastatic nodules.

With the knowledge of the spatiotemporal pattern of metastasis formation in the different cell line derived tumor models of spontaneous metastasis reported here, along with the reported strengths and limitations of the different imaging modalities, we can better plan and perform longitudinal therapeutic response studies leveraging non-invasive assessment of metastatic disease response in the same animals over time in an effort to help advance new anticancer drugs targeted to advanced metastatic disease in both, TN and HER2^+^ breast cancer.

## Conclusions

Non-invasive imaging can be used to assess the spatiotemporal pattern of metastasis formation, overcoming major disadvantages of pure endpoint studies and enables accurate randomization of animal groups for investigating treatment outcome. Our results suggest that [^18^F]FDG-PET and liposome contrast enhanced CT can complement BLI by providing greater sensitivity and 3D spatial information on metastatic disease burden. The described tumor models of spontaneous metastasis and the imaging toolset can be used to assess the efficacy of new therapy regimens for metastatic breast cancer.

## Supporting information

S1 Fig Detailed experimental schedule. (A) In a first set of animals, LM2-4 (*n* = 10) and LM2-4H2N (*n* = 10) cells were inoculated and the primary MFP tumor was resected at 14 days post inoculation. BLI was used to monitor primary tumor growth and metastasis formation, while native CT was used to assess primary tumor growth. 48h prior to study endpoint, the dual-modality CT/fluorescence contrast agent was administered, and CT images were acquired before and 3, 24, and 48h post injection. Fluorescence images were acquired after the animals were sacrificed. (B) LM2-4 (*n* = 7) and LM2-4H2N (*n* = 7) cells were inoculated and the primary MFP tumor was resected at 14 days post inoculation. BLI was used to monitor primary tumor growth and metastasis formation. Metabolic activity of primary tumors and metastasis was assessed using [^18^F]FDG-PET. (C) MDA-MB-231 (*n* = 5) cells were inoculated and the primary MFP tumor was resected at 16 days post inoculation. BLI was used to monitor primary tumor growth and metastasis formation. Native CT was used to assess primary tumor size prior to surgery.
(TIF)

S2 Fig BLI signal ratio metastatic burden/primary tumor after removal of the primary LM2-4 and LM2-4H2N tumor. A significantly increased BLI signal ratio metastatic burden/primary tumor was observed for LM2-4 on day 21 compared to day 7.5 post primary tumor resection (day 7.5: 0.003 ± 0.004, *n* = 12; day 21: 0.127 ± 0.143, *n* = 10; *P* = 0.021, *t*-test), while no difference was found for LM2-4H2N in the same time interval (day 7.5: 0.006 ± 0.007, *n* = 11; day 21: 0.151 ± 0.217, *n* = 10; *P* = 0.059, *t*-test). *P < 0.05, *t*-test.
(TIF)

S3 Fig Biodistribution and kinetics of contrast enhancement following administration of CF800. Whole body CT images before and at 3, 24, and 48h post injection of the contrast agent. P: primary tumor regrowth, L: left.
(TIF)

S1 Appendix Detailed study data. (PDF)
