# Supplementary figures and images for: Spatiotemporal assessment of spontaneous metastasis formation using multimodal in vivo imaging in HER2+ and triple negative metastatic breast cancer xenograft models in mice
Source: PLoS One. 2018 May 3;13(5):e0196892. doi: 10.1371/journal.pone.0196892 (PMC5933713; doi:10.1371/journal.pone.0196892)

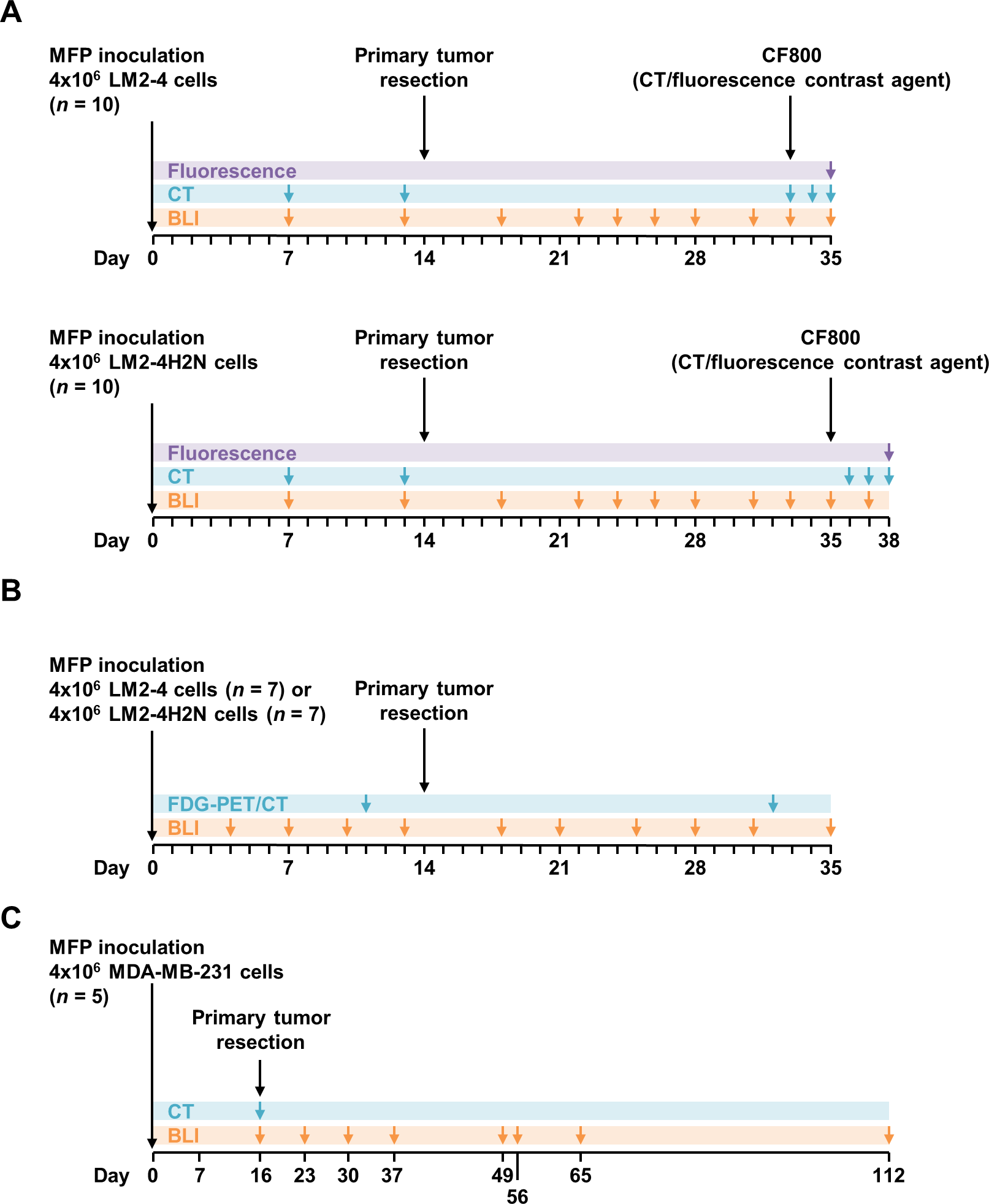

Supplement: S1 Fig — (A) In a first set of animals, LM2-4 (n = 10) and LM2-4H2N (n = 10) cells were inoculated and the primary MFP tumor was resected at 14 days post inoculation. BLI was used to monitor primary tumor growth and metastasis formation, while native CT was used to assess primary tumor growth. 48h prior to study endpoint, the dual-modality CT/fluorescence contrast agent was administered, and CT images were acquired before and 3, 24, and 48h post injection. Fluorescence images were acquired after the animals were sacrificed. (B) LM2-4 (n = 7) and LM2-4H2N (n = 7) cells were inoculated and the primary MFP tumor was resected at 14 days post inoculation. BLI was used to monitor primary tumor growth and metastasis formation. Metabolic activity of primary tumors and metastasis was assessed using [18F]FDG-PET. (C) MDA-MB-231 (n = 5) cells were inoculated and the primary MFP tumor was resected at 16 days post inoculation. BLI was used to monitor primary tumor growth and metastasis formation. Native CT was used to assess primary tumor size prior to surgery. (TIF) [file pone.0196892.s001.tif]

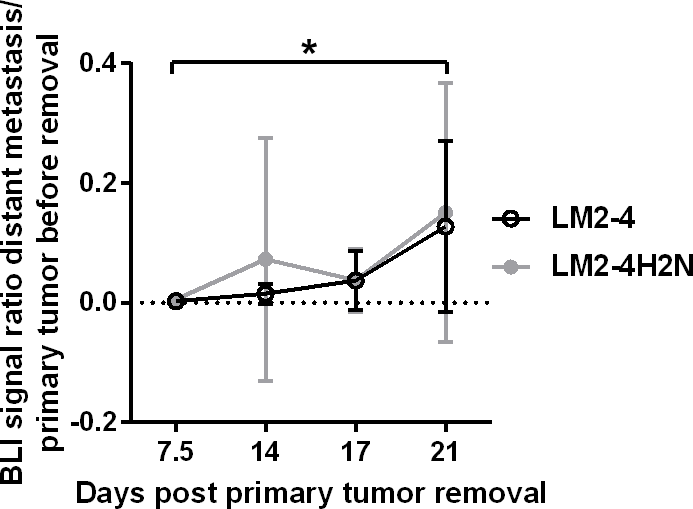

Supplement: S2 Fig — A significantly increased BLI signal ratio metastatic burden/primary tumor was observed for LM2-4 on day 21 compared to day 7.5 post primary tumor resection (day 7.5: 0.003 ± 0.004, n = 12; day 21: 0.127 ± 0.143, n = 10; P = 0.021, t-test), while no difference was found for LM2-4H2N in the same time interval (day 7.5: 0.006 ± 0.007, n = 11; day 21: 0.151 ± 0.217, n = 10; P = 0.059, t-test). *P < 0.05, t-test. (TIF) [file pone.0196892.s002.tif]

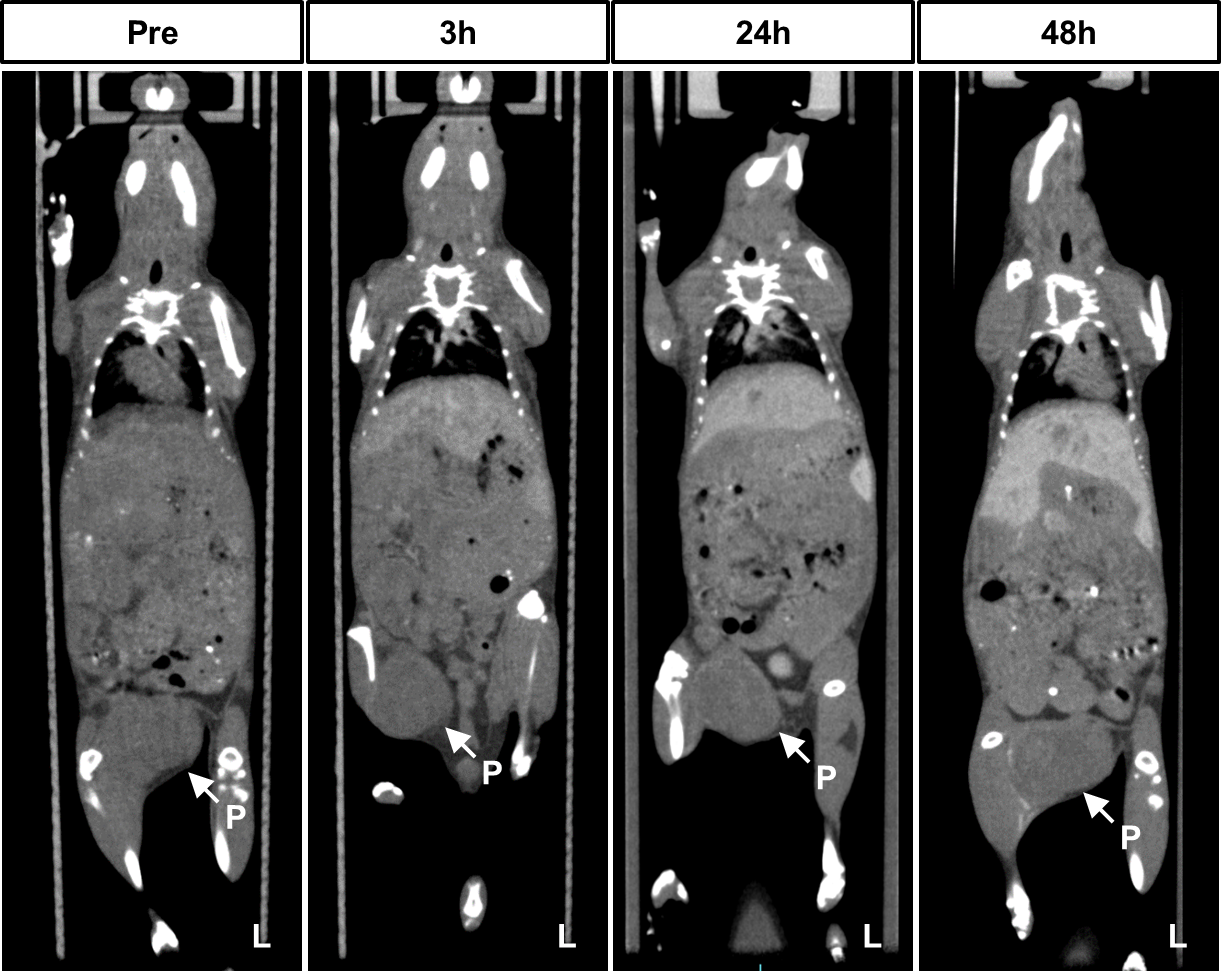

Supplement: S3 Fig — Whole body CT images before and at 3, 24, and 48h post injection of the contrast agent. P: primary tumor regrowth, L: left. (TIF) [file pone.0196892.s003.tif]
