# Supplementary material for: Spatiotemporal assessment of spontaneous metastasis formation using multimodal in vivo imaging in HER2+ and triple negative metastatic breast cancer xenograft models in mice
Source: PLoS One. 2018 May 3;13(5):e0196892. doi: 10.1371/journal.pone.0196892 (PMC5933713; doi:10.1371/journal.pone.0196892)
Supplement: S1 Appendix — (PDF) [file pone.0196892.s004.pdf]

Spatiotemporal assessment of spontaneous metastasis formation using multimodal *in vivo* imaging in HER2$^+$ and triple negative metastatic breast cancer xenograft models in mice

S1 Appendix. Detailed study data.

Inga B. Fricke[1,2,*], Raquel De Souza[1,3], Lais Costa Ayub[3], Giulio Francia[4,#a], Robert Kerbel[4], David A. Jaffray[1,2,5], Jinzi Zheng[1,2]

[1] TECHNA Institute for the Advancement of Technology for Health, University Health Network, Toronto, Ontario, Canada.

[2] Institute of Biomaterials and Biomedical Engineering, University of Toronto, Toronto, Ontario, Canada.

[3] Leslie Dan Faculty of Pharmacy, University of Toronto, Toronto, Ontario, Canada.

[4] Biological Sciences Platform, Sunnybrook Research Institute, Department of Medical Biophysics, University of Toronto, Toronto, Ontario, Canada.

[5] Radiation Medicine Program, Princess Margaret Cancer Centre, Toronto, Ontario, Canada.

[#a] Current address: Border Biomedical Research Center, University of Texas at El Paso, El Paso, Texas, USA.

* Corresponding author: E-mail: Inga.Fricke@rmp.uhn.ca (IBF)

S1 Table. Primary tumor volumes [mm^3] and BLI signal [p/s].

		Caliper Volume [mm^3]	CT Volume [mm^3]	BLI Signal [p/s]
LM2-4 d14	Mouse 1	307.17	297.9	828000000
	Mouse 2	254.4	231.32	488000000
	Mouse 3	216.61	224.18	1440000000
	Mouse 4	246.3	175.84	1260000000
	Mouse 5	335.7	259.69	1800000000
	Mouse 6	307.88	370.16	647000000
	Mouse 7	237.69	252.15	406000000
	Mouse 8	137.16	131.52	508000000
	Mouse 9	290.43	272.03	1030000000
	Mouse 10	425.98	331.7	1550000000
	Mouse 11	214.47	366.7	3280000000
	Mouse 12	368.57	470.6	1830000000
	Mouse 13	267.95	307.2	3940000000
	Mouse 14	417.48	560.6	4500000000
	Mouse 16	236.82	344.1	4240000000
	Mouse 17	391.16	456.9	1560000000
LM2-4H2N d14	Mouse 1	257.67	289.4	426000000
	Mouse 2	264.57	257.33	207000000
	Mouse 3	371.1	339.4	523000000
	Mouse 4	181.19	149.78	307000000
	Mouse 5	248.87	206.47	563000000
	Mouse 6	420.38	348.49	612000000
	Mouse 7	293.07	236.36	441000000
	Mouse 8	177.9	145.6	599000000
	Mouse 9	642.6	434.87	405000000
	Mouse 10	297.8	298.29	510000000
	Mouse 11	385.6	332.5	518000000
	Mouse 12	285.61	220.3	615000000
	Mouse 13	152.81	256.1	356000000
	Mouse 14	211.54	277.2	473000000
	Mouse 16	294.92	315.7	10000000
	Mouse 17	271.43	230.5	1380000000
MDA-MB-231 d16	Mouse 1	70.67	204.33	13200000000
	Mouse 2	163.99	328.18	9930000000
	Mouse 3	543.67	456.73	14100000000
	Mouse 4	354.67	474.61	5170000000
	Mouse 5	388.39	394.66	9990000000

Tumor volumes were determined by Caliper measurements and CT. Measurements were performed on day 14 (LM2-4, LM2-4H2N) or day 16 (MDA-MB-231) post tumor inoculation.

S2 Table. Timing of primary tumor regrowth and formation of first metastasis.

		Primary tumor regrowth	Left inguinal	Liver/ Lung	Right axilla	Left axilla	Other
**LM2-4**	**Mouse 1**	4 dps	-	-	-	-	12 dps
	**Mouse 2**	4 dps	19 dps	14 dps	14 dps	-	-
	**Mouse 3**	4 dps	21 dps	10 dps	10 dps	-	-
	**Mouse 4**	4 dps	-	-	4 dps	-	-
	**Mouse 5**	4 dps	-	-	14 dps	-	-
	**Mouse 6**	4 dps	X	X	X	X	X
	**Mouse 7**	4 dps	8 dps	14 dps	12 dps	19 dps	-
	**Mouse 8**	4 dps	A	A	A	A	A
	**Mouse 9**	4 dps	-	-	-	-	-
	**Mouse 10**	4 dps	8 dps	-	4 dps	-	-
	**Mouse 11**	4 dps	-	4 dps	11 dps	11 dps	-
	**Mouse 12**	7 dps	-	4 dps	11 dps	14 dps	-
	**Mouse 13**	-	-	17 dps	-	-	-
	**Mouse 14**	-	-	-	-	-	-
	**Mouse 15**	X	X	X	X	X	X
	**Mouse 16**	4 dps	-	-	21 dps	-	-
	**Mouse 17**	4 dps	11 dps	21 dps	21 dps	-	-
**LM2-4H2N**	**Mouse 1**	4 dps	12 dps	-	21 dps	-	-
	**Mouse 2**	-	-	-	-	-	-
	**Mouse 3**	4 dps	12 dps	10 dps	8 dps	14 dps	-
	**Mouse 4**	-	-	-	-	-	-
	**Mouse 5**	4 dps	4 dps	4 dps	-	-	-
	**Mouse 6**	4 dps	12 dps	-	17 dps	21 dps	-
	**Mouse 7**	A	A	A	A	A	A
	**Mouse 8**	4 dps	14 dps	19 dps	-	-	-
	**Mouse 9**	4 dps	14 dps	21 dps	21 dps	-	-
	**Mouse 10**	4 dps	17 dps	14 dps	12 dps	14 dps	-
	**Mouse 11**	4 dps	11 dps	21 dps	21 dps	21 dps	-
	**Mouse 12**	4 dps	11 dps	-	-	-	-
	**Mouse 13**	17 dps	-	-	0 dps	0 dps	-
	**Mouse 14**	17 dps	-	-	-	-	-
	**Mouse 15**	-	-	-	-	-	-
	**Mouse 16**	4 dps	-	-	-	-	-
	**Mouse 17**	4 dps	21 dps	-	-	-	-
**MDA-MB-231**	**Mouse 1**	7 dps	36 dps	36 dps	36 dps	-	-
	**Mouse 2**	7 dps	-	36 dps	-	-	21 dps
	**Mouse 3**	7 dps	-	96 dps	-	-	-
	**Mouse 4**	7 dps	-	0 dps	-	-	-

		Primary tumor regrowth	Left inguinal	Liver/ Lung	Right axilla	Left axilla	Other
**MDA-MB-231**	**Mouse 5**	7 dps	7 dps	7 dps	-	-	-

Abbreviations: dps: days post surgery (primary tumor removal); X: animal died; A: animal developed ascites.

S3 Table. Metastatic incidences excluding primary regrowth detected by BLI over time after removal of the primary tumor.

	Number of animals with at least one metastasis		
**Days post primary removal**	**LM2-4**	**LM2-4H2N**	**MDA-MB-231**
0	0/14 (0.00%)	1/16 (6.25%)	1/5 (20.00%)
4	4/14 (28.57%)	2/16 (12.50%)	1/5 (20.00%)
7	4/14 (28.57%)	2/16 (12.50%)	2/5 (40.00%)
8	5/14 (35.71%)	3/16 (18.75%)	2/5 (40.00%)
10	6/14 (42.86%)	3/16 (18.75%)	2/5 (40.00%)
11	7/14 (50.00%)	5/16 (31.25%)	2/5 (40.00%)
12	8/14 (57.14%)	8/16 (50.00%)	2/5 (40.00%)
14	10/14 (71.43%)	10/16 (62.50%)	2/5 (40.00%)
17	11/14 (78.57%)	10/16 (62.50%)	2/5 (40.00%)
21	12/14 (85.71%)	11/16 (68.75%)	3/5 (60.00%)
36	-	-	4/5 (80.00%)
96	-	-	5/5 (100.00%)

S4 Table. Average and median time to develop a first metastasis in the LM2-4, LM2-4H2N and MDA-MB-231 model.

	Time to develop first metastasis (days post primary tumor removal)		
	**LM2-4**	**LM2-4H2N**	**MDA-MB-231**
**Average ± SD**	10 ± 6	11 ± 5	32 ± 38
**Median**	10.5	12	21

S5 Table. Metastatic incidences detected by BLI on day 21 post primary tumor resection for different anatomical locations.

	**Primary tumor regrowth**	**Left inguinal**	**Liver/Lung**	**Right axilla**	**Left axilla**
**LM2-4**	11/12 (91.67%)	5/12 (41.67%)	7/12 (58.33%)	10/12 (83.33%)	3/12 (25.00%)
**LM2-4H2N**	11/11 (100%)	10/11 (90.91%)	6/11 (54.55%)	7/11 (63.64%)	5/11 (45.45%)
**MDA-MB-231**	5/5 (100%)	1/5 (20.00%)	2/5 (40.00%)	0/5 (0.00%)	0/5 (0.00%)

S6 Table. Whole body BLI photon flux [p/s].

	Days post primary tumor removal	-0.5	7.5	14	17	21
LM2-4	Mouse 1	833400000	4186000	77760000	581700000	1516000000
	Mouse 2	492400000	293600000	1475000000	2076000000	1851000000
	Mouse 3	1450000000	506500000	2050000000	2393000000	2211000000
	Mouse 4	1267000000	34520000	219200000	409700000	660000000
	Mouse 5	1805000000	2612000	5593000	6247000	64390000
	Mouse 7	408200000	168600000	2044000000	840100000	1395000000
	Mouse 10	1556000000	1806000000	X	X	X
	Mouse 11	3305000000	33670000	640100000	1459000000	X
	Mouse 12	1857000000	19120000	203600000	326400000	583000000
	Mouse 13	3961000000	816400	3374000	6117000	28220000
	Mouse 16	4274000000	6051000	83310000	301000000	1547000000
	Mouse 17	1580000000	585100000	1651000000	2340000000	5571000000
LM2-4H2N	Mouse 1	429600000	9898000	242800000	857100000	1654000000
	Mouse 3	525700000	247200000	1714000000	4688000000	3373000000
	Mouse 5	568100000	41670000	850800000	62910000	X
	Mouse 6	615300000	416900000	218500000	479000000	1334000000
	Mouse 8	602000000	6802000	74720000	123400000	724500000
	Mouse 9	408600000	198500000	994200000	3890000000	3174000000
	Mouse 10	513100000	894600000	1406000000	4417000000	8920000000
	Mouse 11	519900000	150300000	7903000	1049000000	8169000000
	Mouse 12	617800000	19740000	278100000	234400000	24150000
	Mouse 13	359400000	2461000	3168000	3176000	9630000
	Mouse 17	1386000000	58250000	1157000000	517100000	1894000000
Mean	LM2-4	1899083333	288397950	768448818	976296727	1542661000
	LM2-4H2N	595045455	186029182	631562818	1483735091	2927628000
SD	LM2-4	1280513089	520473486	854310950	925473344	1597590367
	LM2-4H2N	276069757	268739454	613814062	1865721267	3173998410
n =	LM2-4	12	12	11	11	10
	LM2-4H2N	11	11	11	11	10

Abbreviations: X: animal died.

S7 Table. BLI photon flux [p/s] for the primary tumor/primary tumor regrowth.

	Days post primary tumor removal	-0.5	7.5	14	17	21
LM2-4	Mouse 1	827600000	3352000	67610000	578000000	1188000000
	Mouse 2	487500000	291200000	1454000000	2012000000	1705000000
	Mouse 3	1438000000	502600000	2035000000	2374000000	2173000000
	Mouse 4	1257000000	32500000	211400000	384300000	574500000
	Mouse 5	1798000000	1337000	3196000	5403000	61540000
	Mouse 7	405500000	166600000	2023000000	824400000	1318000000
	Mouse 10	1547000000	1781000000	X	X	X
	Mouse 11	3282000000	31770000	567400000	1016000000	X
	Mouse 12	1834000000	18390000	188300000	254400000	153400000
	Mouse 13	3940000000	222600	167500	975500	11600000
	Mouse 16	4240000000	4455000	78820000	293100000	1526000000
	Mouse 17	1557000000	582300000	1637000000	2301000000	5495000000
LM2-4H2N	Mouse 1	426000000	8899000	240100000	851600000	1641000000
	Mouse 3	523400000	245200000	1699000000	4634000000	3295000000
	Mouse 5	563400000	26620000	465300000	61370000	X
	Mouse 6	611500000	413000000	214500000	472300000	1320000000
	Mouse 8	599200000	5668000	72780000	121300000	637300000
	Mouse 9	405200000	197100000	985400000	3850000000	3143000000
	Mouse 10	510300000	889200000	1389000000	4340000000	8561000000
	Mouse 11	517500000	148900000	6984000	1040000000	8001000000
	Mouse 12	615000000	18600000	275100000	231400000	14630000
	Mouse 13	356400000	501100	1507000	1502000	4478000
	Mouse 17	1380000000	58920000	1151000000	510700000	1859000000
Mean	LM2-4	1884466667	284643883	751444864	913052591	1420604000
	LM2-4H2N	591627273	182964373	590970091	1464924727	2847640800
SD	LM2-4	1272323138	514018488	850244632	902918855	1615118478
	LM2-4H2N	275247202	268102675	605906293	1840889445	3077682428
n =	LM2-4	12	12	11	11	10
	LM2-4H2N	11	11	11	11	10

Abbreviations: X: animal died.

S8 Table. Metastatic burden BLI signal [p/s].

	Days post primary tumor removal	-0.5	7.5	14	17	21
**LM2-4**	**Mouse 1**	5800000	834000	10150000	3700000	328000000
	**Mouse 2**	4900000	2400000	21000000	64000000	146000000
	**Mouse 3**	12000000	3900000	15000000	19000000	38000000
	**Mouse 4**	10000000	2020000	7800000	25400000	85500000
	**Mouse 5**	7000000	1275000	2397000	844000	2850000
	**Mouse 7**	2700000	2000000	21000000	15700000	77000000
	**Mouse 10**	9000000	25000000	X	X	X
	**Mouse 11**	23000000	1900000	72700000	443000000	X
	**Mouse 12**	23000000	730000	15300000	72000000	429600000
	**Mouse 13**	21000000	593800	3206500	5141500	16620000
	**Mouse 16**	34000000	1596000	4490000	7900000	21000000
	**Mouse 17**	23000000	2800000	14000000	39000000	76000000
**LM2-4H2N**	**Mouse 1**	3600000	999000	2700000	5500000	13000000
	**Mouse 3**	2300000	2000000	15000000	54000000	78000000
	**Mouse 5**	4700000	15050000	385500000	1540000	X
	**Mouse 6**	3800000	3900000	4000000	6700000	14000000
	**Mouse 8**	2800000	1134000	1940000	2100000	87200000
	**Mouse 9**	3400000	1400000	8800000	40000000	31000000
	**Mouse 10**	2800000	5400000	17000000	77000000	359000000
	**Mouse 11**	2400000	1400000	919000	9000000	168000000
	**Mouse 12**	2800000	1140000	3000000	3000000	9520000
	**Mouse 13**	3000000	1959900	1661000	1674000	5152000
	**Mouse 17**	6000000	-670000	6000000	6400000	35000000
**Mean**	**LM2-4**	14616667	3754067	17003955	63244136	122057000
	**LM2-4H2N**	3418182	3064809	40592727	18810364	79987200
**SD**	**LM2-4**	9812685	6756398	19600895	128221632	143628241
	**LM2-4H2N**	1099835	4281852	114521562	26016449	110293292
***n* =**	**LM2-4**	12	12	11	11	10
	**LM2-4H2N**	11	11	11	11	10

Abbreviations: X: animal died.

S9 Table. [^{18}F]FDG uptake [SUV_{max}] in primary tumor, muscle, and lung before primary tumor resection.

		3 days before primary tumor resection					
		Primary tumor		Muscle		Lung	
		SUV_{max}	Volume [mm^3]	SUV_{max}	Volume [mm^3]	SUV_{max}	Volume [mm^3]
**LM2-4**	**Mouse 11**	3.2780	181.3	0.4549	20.3	0.0046	20.3
	**Mouse 12**	2.4732	259.4	0.6918	20.5	0.0052	20.8
	**Mouse 13**	2.5080	157.7	1.2577	19.8	0.0056	20.2
	**Mouse 14**	3.3853	283.5	0.5683	17.9	0.0044	20.0
	**Mouse 16**	2.3181	174.9	0.1657	19.4	0.0038	20.5
	**Mouse 17**	3.2381	248.3	0.5136	19.6	0.0036	20.3
**LM2-4H2N**	**Mouse 11**	3.5974	201.4	0.5910	20.5	0.0044	20.1
	**Mouse 12**	3.6582	94.1	0.2710	19.8	0.0045	20.3
	**Mouse 13**	3.0982	145.3	0.3176	20.3	0.0039	19.8
	**Mouse 14**	2.5654	161.9	0.2691	20.2	0.0036	20.5
	**Mouse 16**	2.3101	170.2	0.2168	20.0	0.0040	20.1
	**Mouse 17**	1.1936	180.5	0.4086	20.1	0.0035	20.5

[^{18}F]FDG uptake [SUV_{max}] and the volume of the VOI (volume of interest) in mm^3 are listed.

S10 Table. $[^{18}F]$FDG uptake [SUV_{max}] in lung and metastatic lesions after primary tumor removal.

		18 days post primary tumor resection											
		Lung		Lesion 1		Lesion 2		Lesion 3		Lesion 4		Lesion 5	
		SUV_{max}	Size [mm^3]	SUV_{max}	Size [mm^3]	SUV_{max}	Size [mm^3]	SUV_{max}	Size [mm^3]	SUV_{max}	Size [mm^3]	SUV_{max}	Size [mm^3]
**LM2-4**	**Mouse 11**	2.6524	20.1	2.9824[MA]	199.8	2.9717[LA]	203.8	3.1517[LA]	14.7	-	-	-	-
	**Mouse 12**	1.7403	20.0	2.5275[LA]	182.9	2.6860[MA]	85.9	1.3409[MA]	21.3	-	-	-	-
	**Mouse 13**	1.3328	19.8	-	-	-	-	-	-	-	-	-	-
	**Mouse 14**	X	X	X	X	X	X	X	X	X	X	X	X
	**Mouse 16**	0.9225	20.2	3.7914[LA]	40.9	3.2517[LA]	16.8	3.0239[MA]	10.4	-	-	-	-
	**Mouse 17**	0.9646	19.6	3.3387[PTR]	280.1	3.4080[LA]	39.8	4.1309[LA]	157.3	2.7368[MA]	11.3	-	-
**LM2-4H2N**	**Mouse 11**	1.1093	19.9	3.2604[PTR]	260.5	2.6332[MA]	14.6	2.8410[LA]	18.1	3.6800[LA]	25.4	2.1046[MA]	2.5
	**Mouse 12**	0.8655	19.8	3.4327[PTR]	134.5	4.9290[LA]	73.4	2.6008[MA]	6.3	-	-	-	-
	**Mouse 13**	0.8419	20.2	-	-	-	-	-	-	-	-	-	-
	**Mouse 14**	1.4180	19.9	4.0161[MA]	54.3	3.5974[LA]	13.6	3.6147[LA]	10.3	-	-	-	-
	**Mouse 16**	1.4162	20.2	2.4941[LA]	10.5	3.9224[PTR]	365.8	3.2781[LA]	62.9	-		-	-
	**Mouse 17**	1.1088	20.1	3.3090[PTR]	192.3	2.8893[MA]	17.3	3.5121[MA]	18.3	-		-	-

$[^{18}F]$FDG uptake [SUV_{max}] and the volume of the VOI (volume of interest) in mm^3 are listed. X indicates that an animal died prior to image acquisition. Footnotes indicate location of metastatic lesion.

[PTR] primary tumor regrowth

[UA] upper abdomen

[MA] middle abdomen

[LA] lower abdomen
